# Supplementary material for: The Structural Role of RPN10 in the 26S Proteasome and an RPN2-Binding Residue on RPN13 Are Functionally Important in Arabidopsis
Source: Int J Mol Sci. 2024 Oct 30;25(21):11650. doi: 10.3390/ijms252111650 (PMC11546751; doi:10.3390/ijms252111650)
Supplement: Supplementary file 1 [file ijms-25-11650-s001.zip › ijms-3223430-supplementary.pdf]

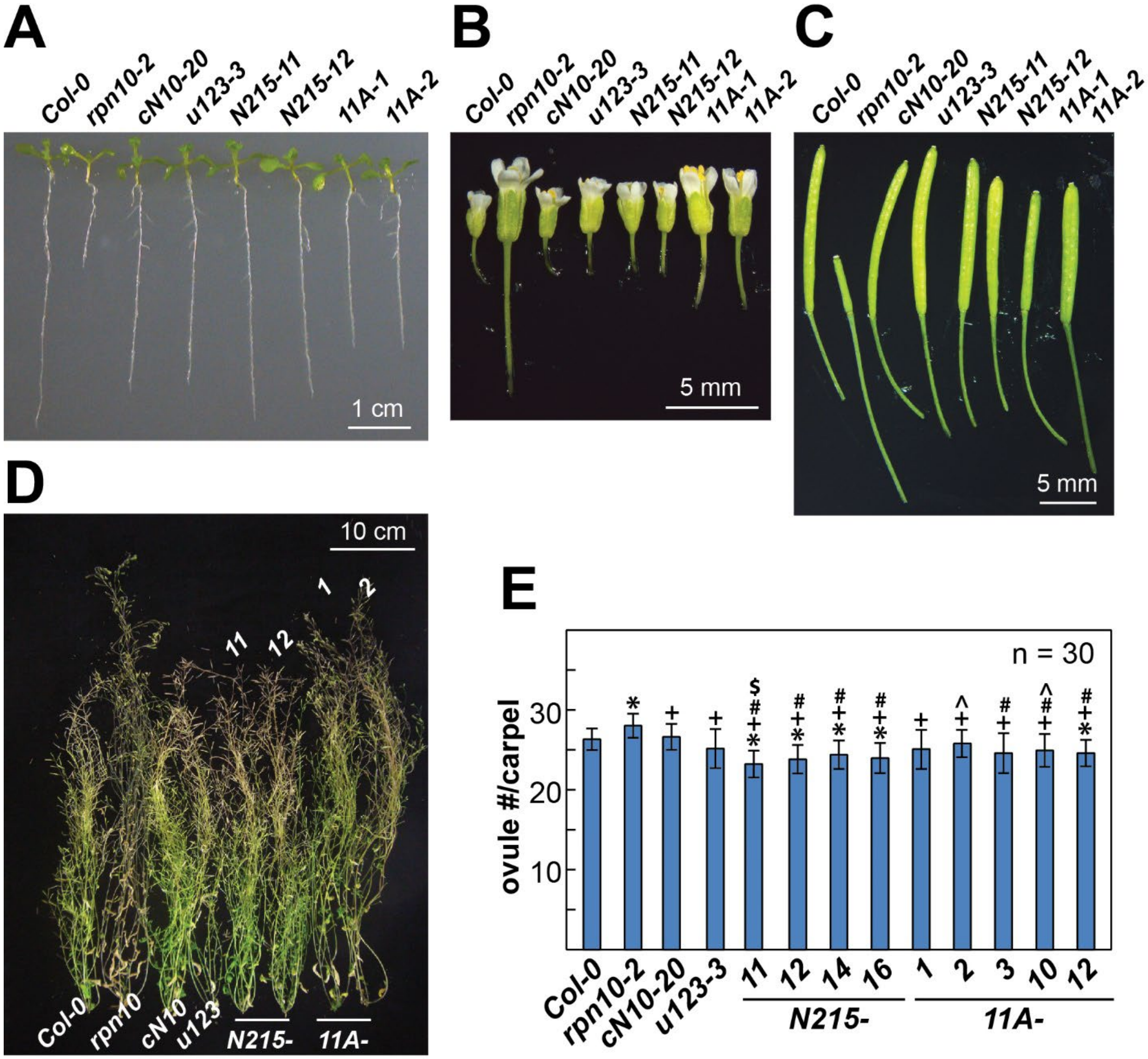

Figure S1. Growth phenotypes for Col-0, *rpn10-2* and various *rpn10-2*-complemented plants. Representative seedlings of 7 DAS **(A)**, flowers **(B)**, siliques **(C),** and 81 DAS plants **(D)** from various lines as designated. **(E)** The average total ovules per carpel from various lines as indicated. Significant difference was determined by pairwise comparison with Col-0 (*, $p < 0.001$), *rpn10-2* (+, $p < 0.0001$), cN10 (#, $p < 0.001$), u123 ($, $p < 0.001$), or N215 (^, $p < 0.001$) using Student's *t* test. The error bars represent the SD.

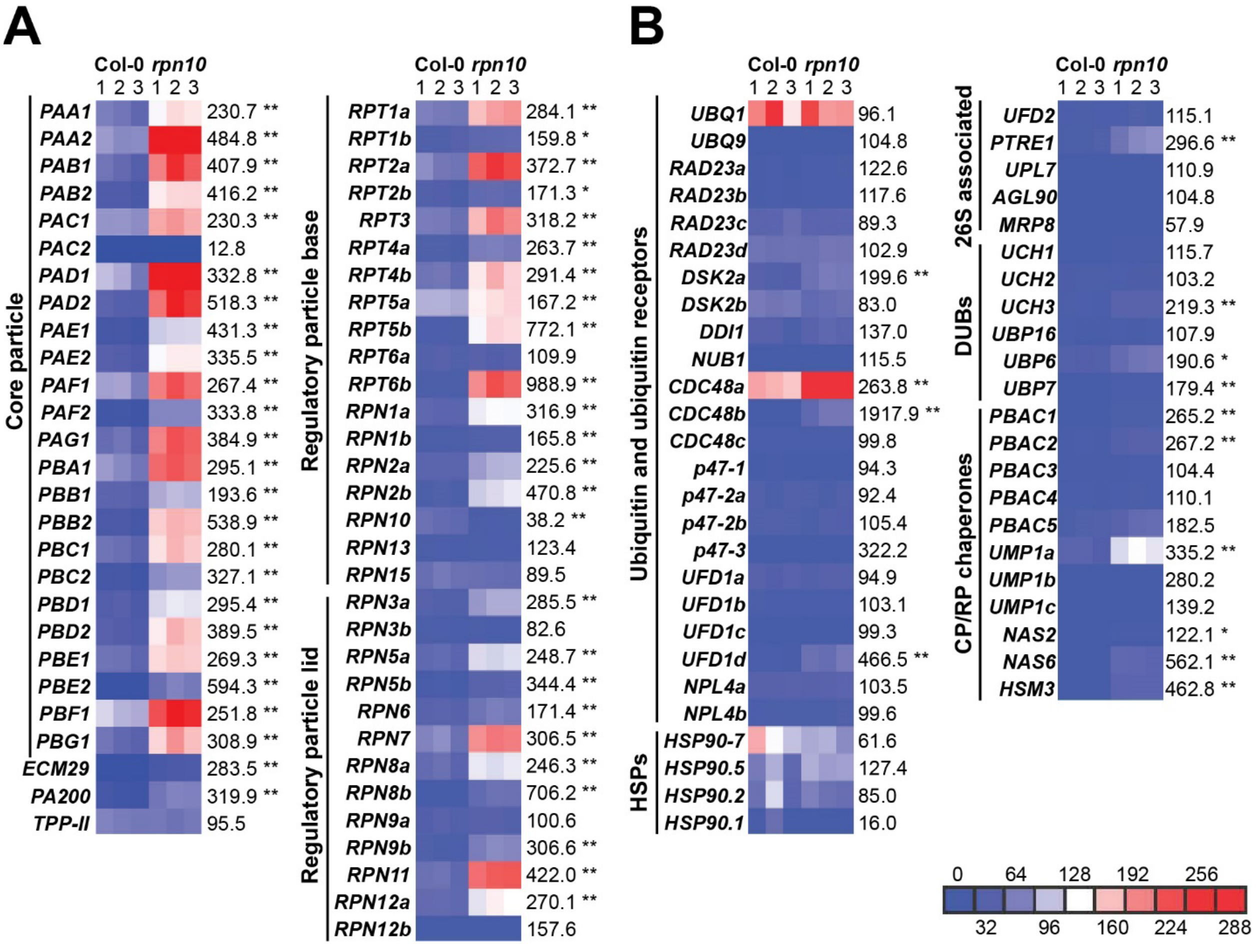

Figure S2. Heat maps showing expression levels for genes encoding 26S proteasome subunits, 26S proteasome-associated proteins, UPS components, and 26S proteasome assembly chaperones. Expression levels (normalized RPKM) for genes encoding core particle subunits, regulatory particle base and lid subunits, ECM29, PA200, TPP-II **(A)**, UPS components including ubiquitin and ubiquitin receptors, 26S proteasome-associated protein, DUBs, heat shock proteins (HSPs), and chaperones involved in CP and RP assembly **(B)** are collected from RNAseq-based transcriptomes of 21 DAS Col-0 and *rpn10-2* rosette leaves (each of three biological repeats 1–3 for Col-0 and *rpn10-2* are shown). Heat maps are generated using the online NGCHM service (Ryan et al., 2019 F1000Res, 8; doi:10.12688/f1000research.20590.2.). Shown to the bottom right is the blue–white–red color gradient with low–middle–high expression levels designated. Numbers to the right of heat maps are averaged percentages of the RPKM for each of examined genes in *rpn10-2* in comparison with that of Col-0; significance determined by Student *t*-test; **, $p < 0.001$; *, $p < 0.005$.

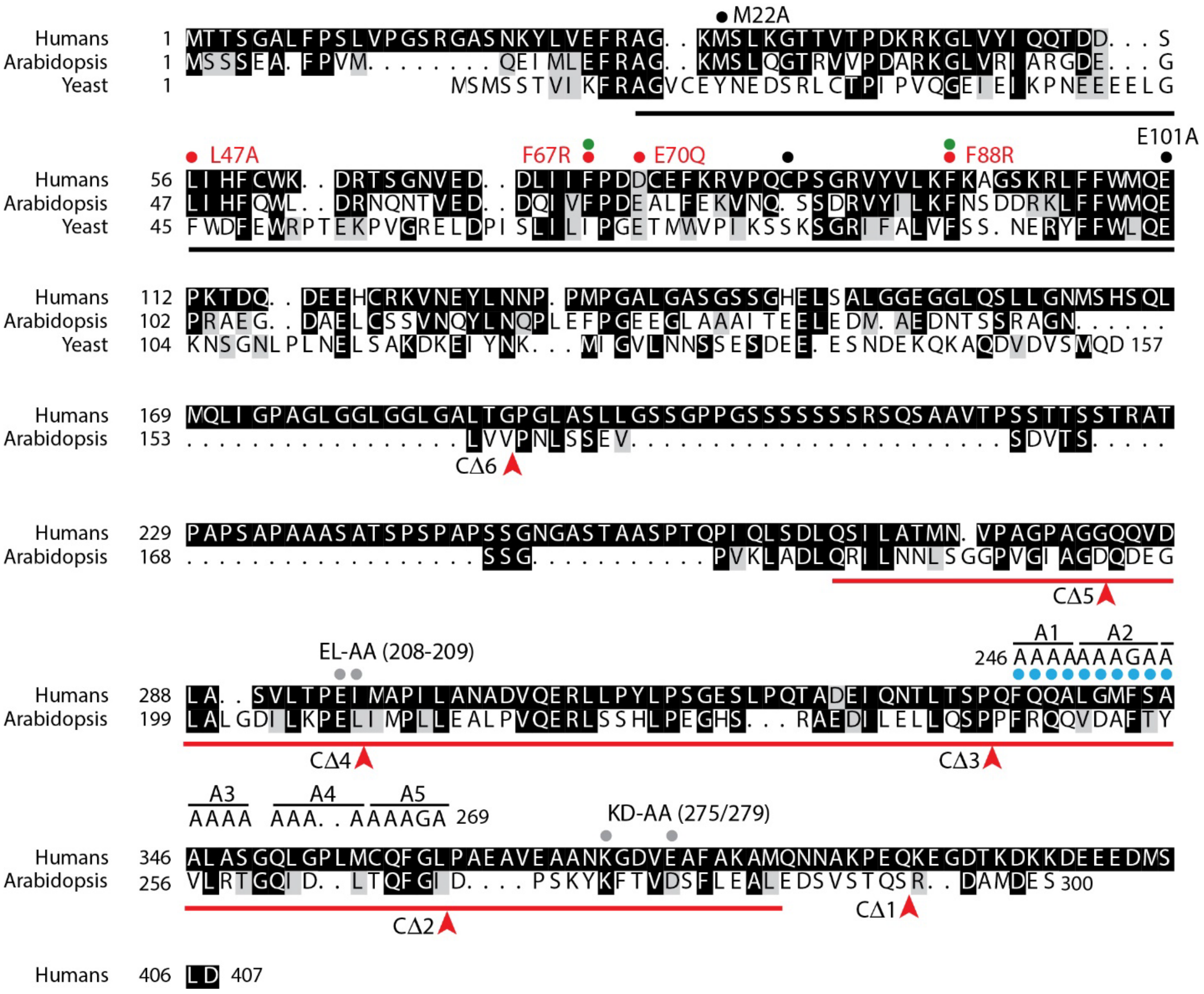

Figure S3. Alignment of protein sequences of RPN13 orthologues from humans, Arabidopsis, and yeast (*Saccharomyces cerevisiae*). The multiple sequence alignment generated by GCG v11.1 (Accelry Inc., San Diego) was subjected to boxshade analysis to display identical and similar residues in reverse types and grey-shaded boxes, respectively, using the Boxshade server (https://junli.netlify.app/apps/boxshade/). The N-terminal PRU and C-terminal DEUBAD domains are marked by thick black and red lines, respectively. Conserved residues M22 and E101 on Arabidopsis RPN13 corresponding to two of three residues M31, C88, and E111 of human RPN13 on the PRU domain shown critical for RPN2 binding by NMR titration are marked in black dots with site-specific mutations tested by Y2H indicated (M22A and E101A). Red dots are the conserved residues on the PRU domain critical for ubiquitin binding for human RPN13 (L56, F76, D79, and F98) and Arabidopsis RPN13 (L47, F67, E70, and F88), which have been confirmed by site-specific mutations (*e.g.*, L47A, F67R, E70Q, and F88R for the

Arabidopsis RPN13, indicated in red fonts next to the red dots) by pull-down assays previously. Two of four residues (F67 and F88), indicated by green dots, on the PRU domain of Arabidopsis RPN13 critical for ubiquitin binding are also involved in RPN2 binding (Figure 3D). Grey dots are conserved residues E208, L209, K275, and D279 on the DEUBAD domain of Arabidopsis RPN13, which have been tested for UCH2 binding by Y2H using site-specific mutations EL-AA (208-209) and KD-AA (275/279), designated next to the grey dots; those residues correspond to some of residues (E295, I296, K371, and E375) critical for UCH37 binding on two regions of the human hRPN13 mapped by NMR and pull-down experiments. The deletion points for serial C-terminal deletions (CΔ1–CΔ6) of the Arabidopsis RPN13 are indicated by red arrowheads. Five all-site-substitution mutations (A1–A5) of subregions between 246–269 of the Arabidopsis RPN13, shown to be critical for UCH2 binding by Y2H, are marked by thin black lines with the substituted residues designated, which were tested in single subregion mutations or serial C-terminally or N-terminally combined subregion mutations (Figure 5). Blue dots marked on residues between 246–254 are critical for UCH2 binding.

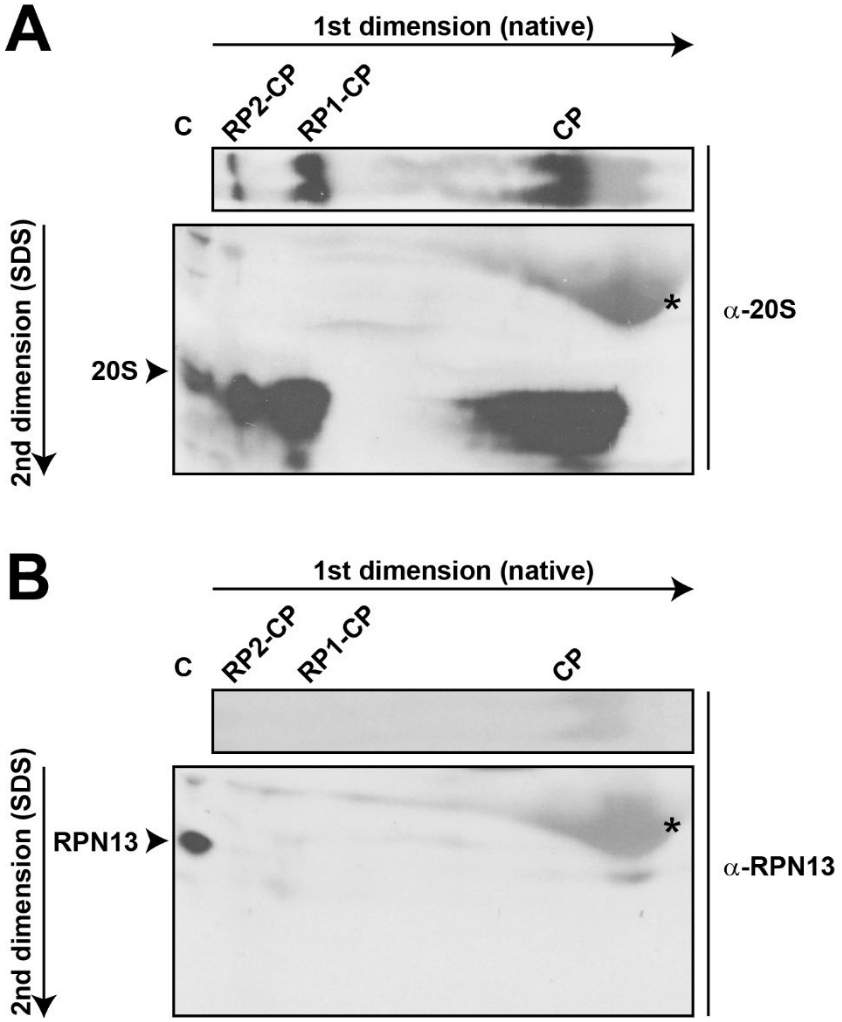

Figure S4. Arabidopsis RPN13 was not found in single- (RP1-CP) and double-capped 20S proteasomes (RP2-CP) in partially purified 26S proteasomes from rosette leaves of Col-0. Approximately 0.8 mg partially purified 26S proteasomes was subjected to a first-dimension native gel electrophoresis followed by a second-dimension SDS-PAGE and immunoblotting using antisera against the moss 20S proteasomes (α-20S). **(A)** or the Arabidopsis RPN13 (α-RPN13) **(B)**. Separate samples of ~200 μg were analyzed only by native gel for comparison (top panels). The mobilized positions for single-, double-capped 20S proteasome, and 20S proteasomes (CP) on the native gels are designated on the top panels. The mobilized positions for 20S proteasome (A) and RPN13 (B) are designated with an arrowhead to the left of the SDS-PAGE gels. C, twenty μg crude extract from rosette leaves of Col-0 was loaded as an antisera control. *, an abundant faint signal detected by both antisera likely represents Rubisco.

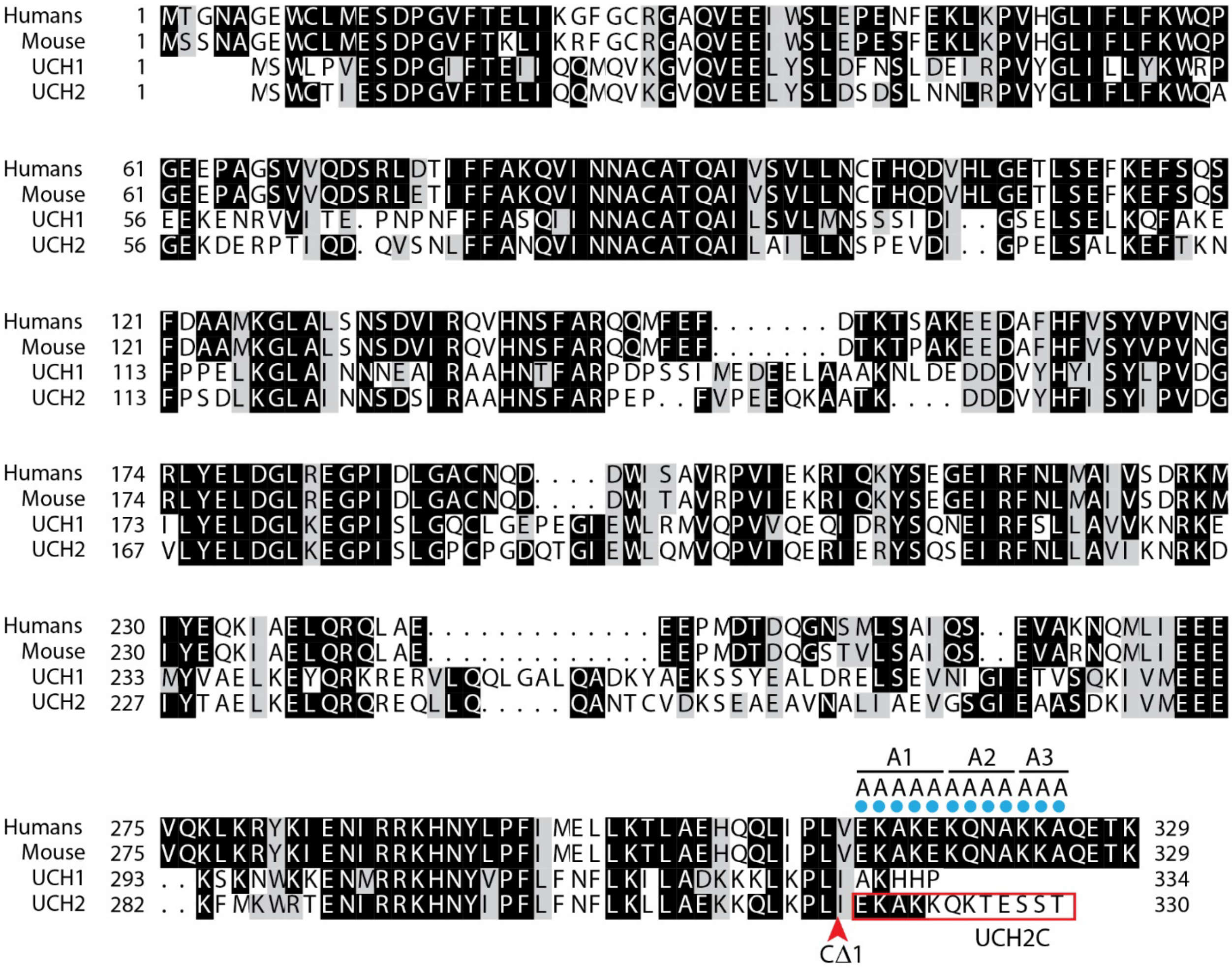

Figure S5. Alignment of protein sequences of Arabidopsis UCH1 and UCH2 with human and mouse UCH37. The multiple sequence alignment generated by GCG v11.1 (Accelry Inc., San Diego) was subjected to boxshade analyses to display identical and similar residues in reverse types and grey-shaded boxes, respectively, using the Boxshade server (https://junli.netlify.app/apps/boxshade/). The unique short highly charged 12-amino acid extension at UCH2 C-terminus is marked by a red rectangle. The deletion point for UCH2-CΔ1 is indicated by a red arrowhead. Three all-alanine-substitution mutations (A1, A2, and A3) on subregions of the 12-amino acid UCH2 C-terminal extension are marked by black lines with substituted residues designated and marked by cyan dots.

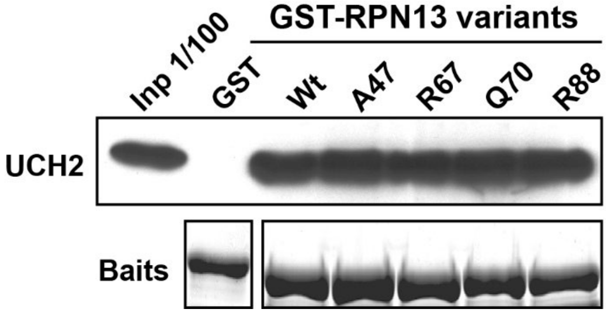

Figure S6. The residues on N-terminal PRU domain of RPN13 critical for ubiquitin and RPN2 binding are not involved in UCH2 interactions. Similar to GST-fused RPN13, all GST-RPN13 variants, which abolished pull-down activity for ubiquitin and/or RPN2, could still readily pull-down UCH2. Amount of prey and baits used in pull-down assays are 5 μg for UCH2, 72 μg for GST, 133 μg for GST-RPN13, and 120 μg for the rest of GST-RPN13 variants. One-hundredth of the input UCH2 (50 ng) and one-tenth of the pulled-down products were analyzed by immunoblotting against α-T7. One-tenth of the pulled-down products (Baits) was examined by staining with Brilliant Blue R to confirm approximately equivalent immobilization. The pulled-down product against GST alone was analyzed as a negative control.

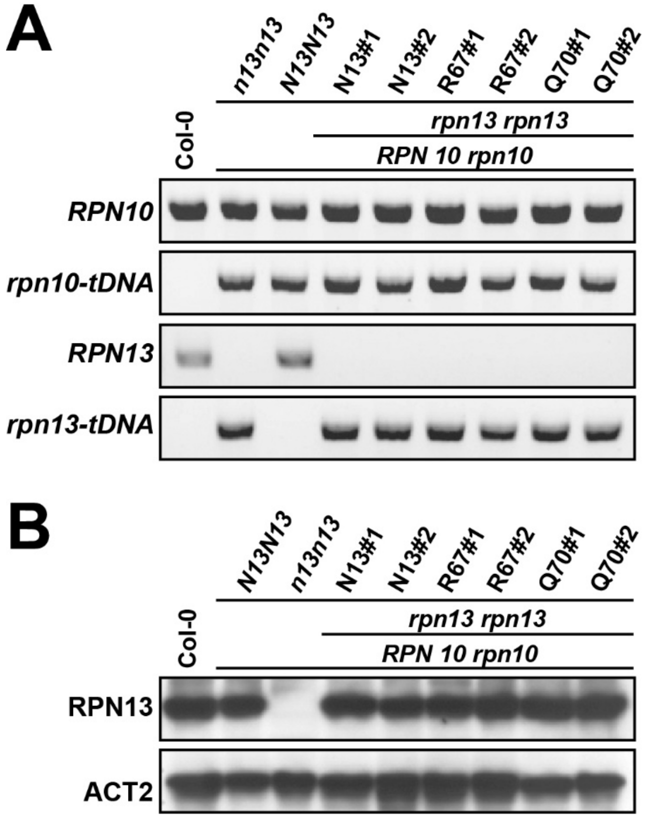

Figure S7. Genotyping and expression of RPN13 variants for the heterozygous *rpn10-2* plants with homozygous *rpn13-1* background harboring each different RPN13 variant. **(A)** The presence of wild-type *RPN10*, T-DNA inserted *rpn10-2* (top two panels), *RPN13* and T-DNA-inserted *rpn13-1* was examined by genomic PCR for Col-0, heterozygous *rpn10-2* in the homozygous *rpn13-1* background (*RPN10 rpn10 rpn13 rpn13*), heterozygous *rpn10-2* in Col-0 background (*RPN10 rpn10 RPN13 RPN13*), and heterozygous *rpn10-2* in the homozygous *rpn13-1* background harboring each of different RPN13 variants (*RPN10 rpn10 rpn13 rpn13* [N13], *RPN10 rpn10 rpn13 rpn13* [R67], and *RPN10 rpn10 rpn13 rpn13* [Q70]). **(B)** Expression of RPN13 was examined by immunoblotting for Col-0, heterozygous *rpn10-2* in Col-0 background (*RPN10 rpn10 RPN13 RPN13*), heterozygous *rpn10-2* in the homozygous *rpn13-1* background (*RPN10 rpn10 rpn13 rpn13*), and heterozygous *rpn10-2* in the homozygous *rpn13-1* background harboring each of different RPN13 variants (*RPN10 rpn10 rpn13 rpn13* [N13], *RPN10 rpn10 rpn13 rpn13* [R67], and *RPN10 rpn10 rpn13 rpn13* [Q70]).

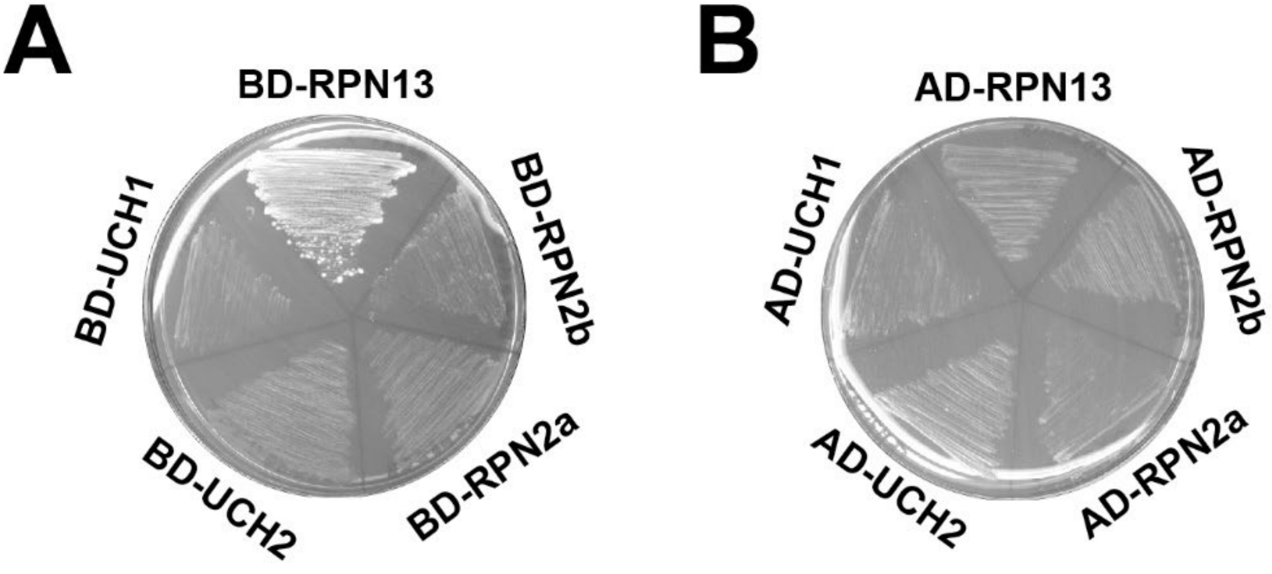

Figure S8. Self-activation was only detected with the BD-fused full-length RPN13. All full-length BD- **(A)** and AD-fusions **(B)** used in this study were tested for self-activation by histidine auxotrophic growth.

Table S1. Distribution of floral organ number (%) for various *rpn10-2*-complemented lines[a].

genotype	Sepal #		Petal #					Stamen #[b]			Carpel#
	4	5	3	4	5	6	7	4	5	6	2
Col-0	100	0	0	100	0	0	0	0	0	100	100
rpn10-2	86	14	4	26	58	10	2	6	64	30	100
cN10-20	100	0	0	100	0	0	0	0	0	100	100
u123-3	100	0	0	100	0	0	0	0	0	100	100
N215-11	100	0	0	100	0	0	0	0	0	100	100
N215-12	100	0	0	100	0	0	0	0	0	100	100
N215-14	100	0	0	100	0	0	0	0	0	100	100
N215-16	100	0	0	100	0	0	0	0	0	100	100
11A-1	100	0	0	92	8	0	0	0	44	56	100
11A-2	100	0	0	98	0	2	0	0	56	44	100
11A-3	100	0	0	98	2	0	0	2	62	36	100
11A-10	100	0	0	100	0	0	0	4	64	32	100
11A-12	100	0	0	100	0	0	0	4	46	50	100

[a]Data were derived from 50 flowers for each genotypes.

[b]Flowers from *Col-0*, *cN10-2*, *u123-3*, and *N215* plants typically have stamens with four long and two short filaments. Stamens from the same flowers in *rpn10-2* plants generally have similar filament lengths. Some of the flowers from *rpn10-2*, *11A-1*, and *11A-2* plants have one shrunken anthers (6%, 2% and 2%, respectively).

Table S2. Expression (RPKM) examined by RNAseq for genes encoding 26S proteasome subunits and associated factors from *rpn10-2*[a].

subunit	locus	*RPKM* *rpn10-2* ± SD/Col-0 ± SD (%)[b]
CP		
PAA1	At5g35590	137.0 ± 12.5/59.4 ± 7.3 (230.7) **
PAA2	At2g05840	358.6 ± 41.3/74.0 ± 4.5 (484.8) **
PAB1	At1g16470	212.9 ± 24.2/52.2 ± 9.7 (407.9) **
PAB2	At1g79210	145.3 ± 5.8/34.9 ± 4.7 (416.2) **
PAC1	At3g22110	173.5 ± 7.0/75.4 ± 2.1 (230.3) **
PAC2	At4g15160	0.1 ± 0.0/0.4 ± 0.2 (12.8)
PAD1	At3g51260	271.3 ± 22.2/81.5 ± 17.6 (332.8) **
PAD2	At5g66140	226.9 ± 27.1/43.8 ± 4.4 (518.3) **
PAE1	At1g53850	105.1 ± 3.2/24.4 ± 3.5 (431.3) **
PAE2	At3g14290	133.6 ± 7.7/39.8 ± 4.9 (335.5) **
PAF1	At5g42790	203.1 ± 13.1/76.0 ± 9.6 (267.4) **
PAF2	At1g47250	69.4 ± 0.5/20.8 ± 3.7 (333.8) **
PAG1	At2g27020	204.5 ± 13.4/53.1 ± 9.5 (384.9) **
PBA1	At4g31300	207.1 ± 6.4/70.2 ± 9.3 (295.1) **
PBB1	At3g27430	90.1 ± 4.1/46.5 ± 3.2 (193.6) **
PBB2	At5g40580	160.4 ± 7.0 /29.8 ± 3.6 (538.9) **
PBC1	At1g21720	154.1 ± 11.6/55.0 ± 6.7 (280.1) **
PBC2	At1g77440	76.0 ± 3.5/23.2 ± 4.2 (327.1) **
PBD1	At3g22630	112.3 ± 4.8/38.0 ± 5.8 (295.4) **
PBD2	At4g14800	153.9 ± 14.4/39.5 ± 4.2 (389.5) **
PBE1	At1g13060	152.0 ± 4.7/56.5 ± 5.9 (269.3) **
PBE2	At3g26340	62.9 ± 5.7/10.6 ± 1.3 (594.3) **
PBF1	At3g60820	237.4 ± 20.4/94.3 ± 11.1 (251.8) **
PBG1	At1g56450	164.3 ± 18.0/53.2 ± 7.6(308.9) **
26S proteasome-associated factors		
ECM29	At2g26780	31.0 ± 2.1/10.9 ± 1.0 (283.5) **
PA200	At3g13330	65.5 ± 3.6/20.5 ± 4.7 (319.9) **
TPP-II	At4g20850	61.9 ± 2.3/64.9 ± 3.0 (95.5)

subunit	locus	*RPKM* *rpn10-2* ± SD/Col-0 ± SD (%)
Base		
RPT1a	At1g53750	173.8 ± 12.3/61.2 ± 2.8 (284.1) **
RPT1b	At1g53780	43.8 ± 0.7/27.4 ± 4.6 (159.8) *
RPT2a	At4g29040	230.3 ± 19.8/61.8 ± 8.1 (372.7) **
RPT2b	At2g20140	45.4 ± 2.5/26.5 ± 5.0 (171.3) *
RPT3	At5g58290	184.8 ± 20.2/58.1 ± 5.2 (318.2) **
RPT4a	At5g43010	62.4 ± 1.5/23.7 ± 2.5 (263.7) **
RPT4b	At1g45000	153.8 ± 15.0/52.8 ± 7.2 (291.4) **
RPT5a	At3g05530	143.8 ± 6.1/86.0 ± 2.9 (167.2) **
RPT5b	At1g09100	139.0 ± 16.3/18.0 ± 1.2 (772.1) **
RPT6a	At5g19990	31.4 ± 1.6/28.6 ± 2.9 (109.9)
RPT6b	At5g20000	207.4 ± 19.2/21.0 ± 3.6 (988.9) **
RPN1a	At2g20580	121.8 ± 4.2/38.4 ± 2.6 (316.9) **
RPN1b	At4g28470	26.5 ± 1.1/16.0 ± 1.0 (165.8) **
RPN2a	At2g32730	85.1 ± 6.3/37.7 ± 0.7 (225.6) **
RPN2b	At1g04810	108.2 ± 7.0/23.0 ± 1.2 (470.8) **
RPN10	At4g38630	18.7 ± 2.2/48.9 ± 4.3 (38.2) **
RPN13	At2g26590	19.5 ± 1.2/15.8 ± 2.5 (123.4)
RPN15	At1g64750	49.3 ± 1.5/55.1 ± 4.1 (89.5)
Lid		
RPN3a	At1g20200	81.9 ± 4.9/28.7 ± 2.7 (285.5) **
RPN3b	At1g75990	14.8 ± 1.4/17.9 ± 4.1 (82.6)
RPN5a	At5g09900	107.8 ± 2.9/43.3 ± 4.3 (248.7) **
RPN5b	At5g64760	36.1 ± 2.4/10.5 ± 1.7 (344.4) **
RPN6	At1g29150	59.6 ± 3.7/34.8 ± 2.0 (171.4) **
RPN7	At4g24820	192.9 ± 6.4/62.9 ± 9.9 (306.5) **
RPN8a	At5g05780	113.1 ± 4.0/45.9 ± 8.2 (246.3) **
RPN8b	At3g11270	51.4 ± 4.5/7.3 ± 0.8 (706.2) **
RPN9a	At5g45620	30.3 ± 2.3/30.2 ± 3.5 (100.6)
RPN9b	At4g19006	66.4 ± 3.4/21.6 ± 3.5 (306.6) **
RPN11	At5g23540	212.8 ± 8.3/50.4 ± 6.4 (422.0) **
RPN12a	At1g64520	125.9 ± 12.7/46.6 ± 1.3 (270.1) **
RPN12b	At5g42040	1.2 ± 0.2/0.7 ± 0.1 (157.6)

[a]Expression (RPKM) for genes encoding subunits of core particle (*CP*), RP base subcomplex (*Base*), RP lid subcomplex (*Lid*), and 26S proteasome-associated proteins ECM29, PA200, and TPP-II were extracted from RNAseq-based transcriptomes established from 21 DAS Col-0 and *rpn10-2* rosette leaves (the complete transcriptomes analyses will be reported elsewhere).

[b]Averaged expression (RPKM) ± SD for each of examined genes in *rpn10-2* vs. Col-0 are shown, numbers in parentheses are percentage of the RPKM for each of examined genes in *rpn10-2* comparing with that of Col-0; significance determined by Student *t*-test; **, $p < 0.001$; *, $p < 0.005$.

Table S3. Expression (RPKM) examined by RNAseq for genes encoding UPS components and major proteasome assembly chaperones from *rpn10-2*[a].

subunit	locus	*RPKM* *rpn10-2* ± SD /Col-0 ± SD (%)[b]
Ubiquitin and ubiquitin receptors		
UBQ1	At3g52590	198.8 ± 28.8/206.9 ± 73.7 (96.1)
UBQ9	At5g37640	0.0 ± 0.0/0.0 ± 0.0 (104.8)
RAD23a	At1g16190	13.2 ± 1.9/10.7 ± 2.4 (122.6)
RAD23b	At1g79650	13.3 ± 0.2/11.3 ± 0.9 (117.6)
RAD23c	At3g02540	38.8 ± 2.0/43.5 ± 7.1 (89.3)
RAD23d	At5g38470	56.0 ± 3.0/54.4 ± 1.9 (102.9)
DSK2a	At2g17190	58.5 ± 3.9/29.3 ± 1.8 (199.6) **
DSk2b	At2g17200	49.3 ± 3.7/59.4 ± 2.1 (83.0)
DDI1	At3g13235	38.8 ± 3.9/28.3 ± 5.7 (137.0)
NUB1	At2g12550	5.3 ± 0.5/4.6 ± 0.2 (115.5)
CDC48a	At3g09840	438.6 ± 30.7/166.3 ± 8.6 (263.8) **
CDC48b	At3g53230	53.2 ± 8.1/2.8 ± 1.4 (1917.9) **
CDC48c	At5g03340	12.9 ± 0.7/12.9 ± 0.5 (99.8)
p47-1	At4g15410	8.0 ± 0.4/8.5 ± 1.1 (94.3)
p47-2a	At4g04210	24.3 ± 2.1/26.3 ± 2.9 (92.4)
p47-2b	At4g22150	27.0 ± 4.7/25.6 ± 1.4 (105.4)
p47-3	At3g21660	0.1 ± 0.1/0.0 ± 0.0 (322.2)
UFD1a	At2g21270	31.4 ± 1.9/33.0 ± 2.5 (94.9)
UFD1b	At4g38930	14.1 ± 0.7/13.7 ± 1.5 (103.1)
UFD1c	At2g29070	7.7 ± 1.6/7.8 ± 1.3 (99.3)
UFD1d	At4g15420	55.0 ± 5.5/11.8 ± 0.7 (466.5) **
NPL4a	At3g63000	34.8 ± 2.7/33.7 ± 1.5 (103.5)
NPL4b	At2g47970	21.5 ± 1.1/21.6 ± 2.2 (99.6)
Heat shock proteins		
HSP90-7	At4g24190	80.6 ± 9.6/130.9 ± 37.8 (61.6)
HSP90.5	At2g04030	81.7 ± 3.9/64.1 ± 20.9 (127.4)
HSP90.2	At5g56030	56.8 ± 6.5/66.8 ± 36.8 (85.0)
HSP90.1	At5g52640	3.5 ± 1.4/22.0 ± 25.5 (16.0)

subunit	locus	*RPKM* *rpn10-2* ± SD /Col-0 ± SD (%)
26S proteasome-associated factors		
UFD2	At5g15400	21.1 ± 1.3/18.3 ± 1.9 (115.1)
PTRE1	At3g53970	65.8 ± 8.1/22.2 ± 1.5 (296.6) **
UPL7	At3g53090	7.7 ± 0.6/6.9 ± 0.8 (110.9)
AGL90	At5g27960	0.0 ± 0.0/0.0 ± 0.0 (104.8)
MRP8	At3g13090	0.1 ± 0.1/0.2 ± 0.1 (57.9)
DUBs and 26S proteasome-associated DUBs		
UCH1	At5g16310	9.8 ± 0.6/8.5 ± 0.5 (115.7)
UCH2	At1g65650	17.7 ± 0.2/17.2 ± 0.5 (103.2)
UCH3	At4g17510	35.1 ± 0.4/16.0 ± 2.2 (219.3) **
UBP16	At4g24560	12.0 ± 0.3/11.1 ± 1.3 (107.9)
UBP6	At1g51710	54.0 ± 5.2/28.3 ± 2.2 (190.6) *
UBP7	At3g21280	13.8 ± 0.8/7.7 ± 0.3 (179.4) **
Core particle assembly chaperones		
PBAC1	At3g25545	22.6 ± 1.1/8.5 ± 0.5 (265.2) **
PBAC2	At3g18940	31.4 ± 1.8/11.7 ± 0.6 (267.2) **
PBAC3	At5g14710	11.2 ± 1.6/10.7 ± 0.6 (104.4)
PBAC4	At1g48170	14.2 ± 2.1/12.9 ± 1.4 (110.1)
PBAC5	At3g07640	44.6 ± 8.0/24.4 ± 2.4 (182.5)
Chaperones for connecting CP halves		
UMP1a	At1g67250	116.8 ± 10.9/34.8 ± 4.5 (335.2) **
UMP1b	At5g38650	0.0 ± 0.0/0.0 ± 0.0 (280.2)
UMP1c	At1g62920	5.2 ± 0.7/3.8 ± 0.6 (139.2)
Regulatory particle assembly chaperones		
NAS2	At5g57950	10.6 ± 0.4/8.6 ± 0.6 (122.1) *
NAS6	At2g03430	42.6 ± 1.2/7.6 ± 0.7 (562.1) **
HSM3	At3g15180	39.4 ± 1.9/8.5 ± 0.7 (462.8) **

[a]Expression (RPKM) for genes encoding ubiquitin, ubiquitin receptors, heat shock proteins, 26S proteasome-associated proteins, DUBs, assembly chaperones for core particle and regulatory particle, and UMP1a–c (Chaperones for connecting CP halves) were extracted from RNAseq-based transcriptomes established from 21 DAS Col-0 and *rpn10-2* rosette leaves (the complete transcriptomes analyses will be reported elsewhere).

[b]Averaged expression (RPKM) ± SD for each of examined genes in *rpn10-2* vs. Col-0 are shown, numbers in parentheses are percentage of the RPKM for each of examined genes in *rpn10-2* comparing with that of Col-0; significance determined by Student *t*-test; **, $p < 0.001$; *, $p < 0.005$.

Table S4. Constructs for yeast-two-hybrid, GST pull-down, and Arabidopsis transformations.

Name[a]	Template or source clone/content	Destination vector, sites[b]	Primer pair for amplification/mutagenesis and/or construction notes
Intermediate *RPN10* constructs for Agrobacterium-mediated transformation			
pBI121-RPN10	First strand cDNAs/*RPN10* coding region (*RPN10*)	pBI121, Sm/Sc	cRPN10-Sma: TCccCggGATGGTTCTCGAGGCGACTATG<br>cRPN10-Sst: ATgagctcTCACTTCTTCTCATCCTCGCC<br>The Arabidopsis *RPN10* coding region was amplified, restricted and cloned into pBI121.
pBI121-RPN10-11A	pGEMT-RPN10-11A /*RPN10-11A*	pBI121, Sm/Sc	The coding region for *RPN10-11A* from pGEMT-RPN10-11A was subloned into pBI121.
pBI121-RPN10-NΔ53	First strand cDNAs/*RPN10-NΔ53*	pBI121, Sm/Sc	NΔ53-Sma: ATT**cccggg**ATGGCTGGCAAAGGAG<br>cRPN10-Sst: ATgagctcTCACTTCTTCTCATCCTCGCC<br>The coding region for *RPN10-NΔ53* was amplified, restricted, and cloned into pBI121.
pKS-pN10	Col-0 genomic DNA/*RPN10* promoter (*pRPN10*)	pBluescript KS+, S/Sm	AtRPN10-1: AAT**gTCGac**TGGGTACTACTGGGCTTCTTCGAGTGTC<br>AtRPN10-6: CAT**CccGggG**ATAAGCAGAGAGAAGCTTCAAAAATTCTGTCG<br>A 2267-bp *RPN10* promoter was amplified, restricted, and cloned into pBluescript KS+.
pKS-pN10-cN10	First strand cDNAs/ *pRPN10-RPN10*	pKS-pN10, Sm/Sc	cRPN10-Sma: TCccCggGATGGTTCTCGAGGCGACTATG<br>cRPN10-Sst: ATgagctcTCACTTCTTCTCATCCTCGCC<br>The *RPN10* coding region was amplified, restricted, and cloned into pKS-pN10.
pKS-pN10-cN10-11A	pBI121-RPN10-11A/*pRPN10-RPN10-11A*	pKS-pN10-cN10, Sm/Sc	The coding region for *RPN10* from pKS-pN10-cN10 was replaced by *RPN10-A11* from pBI121-RPN10-11A.
pKS-pN10-cN10-NΔ15	First strand cDNAs/ *pRPN10-RPN10-NΔ15*	pKS-pN10, Sm/Sc	NΔ15-SmaI: t**cccgGG**ATGCGAAACGGAGATTACTCTC<br>cRPN10-Sst: ATgagctc**TCA**CTTCTTCTCATCCTCGCC<br>The coding region for *RPN10-NΔ15* was amplified, restricted, and cloned into pKS-pN10.
pKS-pN10-cN10-NΔ42	First strand cDNAs/ *pRPN10-RPN10-NΔ42*	pKS-pN10, Sm/Sc	NΔ42-SmaI: t**cccggg**atGTCGAATCCGGAGAATACGGTGGG<br>cRPN10-Sst: ATgagctc**TCA**CTTCTTCTCATCCTCGCC<br>The coding region for *RPN10-NΔ42* was amplified, restricted, and cloned into pKS-pN10.
pKS-pN10-cN10-NΔ53	pBI121-RPN10-NΔ53/*pRPN10-RPN10-NΔ53*	pKS-pN10, Sm/Sc	The coding region for *RPN10-NΔ53* from pBI121-RPN10-NΔ53 was subloned into pKS-pN10.
pKS-pN10-cN10-10-14A5	pGEMT-RPN10-10-14A5/ *pRPN10-RPN10-10-14A5*	pKS-pN10-N10-NΔ42, Sm/Sc	The coding region for *RPN10-NΔ42* from pKS-pN10-cN10-NΔ42 was replaced by *RPN10-10-14A5* from pGEMT-RPN10-10-14A5.
pKS-pN10-cN10-N215	pGEMT-RPN10-N215/ *pRPN10-RPN10-N215*	pKS-pN10-N10-NΔ42, Sm/Sc	The coding region for *RPN10-NΔ42* from pKS-pN10-N10-NΔ42 was replaced by *RPN10-N215* from pGEMT-RPN10-N215.
pGEMT-RPN10	First strand cDNAs/*RPN10* coding region (*RPN10*)	pGEMT	cRPN10-Sma: TCccCggG**ATG**GTTCTCGAGGCGACTATG<br>cRPN10-Sst: ATgagctc**TCA**CTTCTTCTCATCCTCGCC<br>The Arabidopsis *RPN10* coding region was amplified and cloned directly into pGEMT.
pGEMT-RPN10-11A	pGEMT-RPN10/ *RPN10-11A*	pGEMT-RPN10	AtRPN10A11-top: GACTATGATATGTATC**GcC**AACTCCGAGTGGATG<br>AtRPN10A11-bot: CATCCACTCGGAGTT**GgC**GATACATATCATAGTC<br>The D11 codon for *RPN10* was site-specifically mutagenized to a codon for Ala.
pGEMT-RPN10-11K	pGEMT-RPN10/ *RPN10-11K*	pGEMT-RPN10	AtRPN10_D11K_5': GACTATGATATGTATC**aAg**AACTCCGAGTGGATG<br>AtRPN10_D11K_3': CATCCACTCGGAGTT**cTt**GATACATATCATAGTC<br>The D11 codon for *RPN10* was site-specifically mutagenized to a codon for Lys.
pGEMT-RPN10-11R	pGEMT-RPN10/ *RPN10-11R*	pGEMT-RPN10	AtRPN10_D11R_5': GACTATGATATGTATC**cgC**AACTCCGAGTGGATG<br>AtRPN10_D11R_3': CATCCACTCGGAGTT**Gcg**GATACATATCATAGTC<br>The D11 codon for *RPN10* was site-specifically mutagenized to a codon for Arg.
pGEMT-RPN10-11-12RR	pGEMT-RPN10/ *RPN10-11-12RR*	pGEMT-RPN10	AtRPN10_11-12R_5': GACTATGATATGTATC**cgCcgC**TCCGAGTGGATG<br>AtRPN10_11-12R_3': CATCCACTCGGA**GcgGcg**GATACATATCATAGTC<br>The D11 and N12 codons for *RPN10* were both site-specifically mutagenized to a codon for Arg.
pGEMT-RPN10-N215	pGEMT-RPN10/ *RPN10-N215*	pGEMT-RPN10-10-14A5, Sm/Sc	cRPN10-Sma: TCccCggG**ATG**GTTCTCGAGGCGACTATG<br>cRPN10-N215_3' Sst: TCgAgctCtcAGTCGAAGTCCCCACCTGCGGC<br>The coding region for *RPN10-A1014A5* was replaced by the PCR product for *RPN10-N215*.
pGEMT-RPN10-10-14A5	pGEMT-RPN10/ *RPN10-10-14A5*	pGEMT-RPN10	1014Ala_up: GGTTCTCGAGGCGACTATGATATGT**gcgGcggcggCgGcG**TGGATGCGAAACGGAGATTAC<br>1014Ala_down: GTAATCTCCGTTTCGCATCCA**CgCcGccgccgCcgc**ACATATCATAGTCGCCTCGAGAACC<br>The IDNSE (10-14) codons for *RPN10* were all site-specifically mutagenized to codons for Ala.
Final *RPN10* constructs for Agrobacterium-mediated transformation			
ptRPN10	pKS-pN10-cN10/ *pRPN10-RPN10*	pBI101.2, S/Sc	The S/Sc-fragment for *RPN10* promoter and *RPN10* coding region from pKS-pN10-cN10 was subcloned into pBI101.2.
ptRPN10-NΔ15	pKS-pN10-cN10-NΔ15/	pBI101.2, S/Sc	The S/Sc-fragment for *RPN10* promoter and *RPN10-NΔ15* coding region from pKS-pN10-cN10-NΔ15 was subcloned into pBI101.2.

	pRPN10-RPN10-NΔ15		
ptRPN10-NΔ42	pKS-pN10-cN10- NΔ42/ *pRPN10-RPN10-NΔ42*	pBI101.2, S/Sc	The S/Sc-fragment for *RPN10* promoter and *RPN10-NΔ42* coding region from pKS-pN10-cN10-NΔ42 was subcloned into pBI101.2.
ptRPN10-NΔ53	pKS-pN10-cN10- NΔ53/ *pRPN10-RPN10-NΔ53*	pBI101.2, S/Sc	The S/Sc-fragment for *RPN10* promoter and *RPN10-NΔ53* coding region from pKS-pN10-cN10-NΔ53 was subcloned into pBI101.2.
ptRPN10-N215	pGEMT-RPN10-N215/ *pRPN10-RPN10-N215*	ptRPN10, Sm/Sc	The Sm/Sc-fragment for the *RPN10* coding region from ptRPN10 was replaced by the *RPN10-N215* coding region from pGEMT-RPN10-N215.
ptRPN10-11A	pKS-pN10-cN10-11A/ *pRPN10-RPN10-11A*	pBI101.2, S/Sc	The S/Sc-fragment for *RPN10* promoter and *RPN10-11A* coding region from pKS-pN10-cN10-11A was subcloned into pBI101.2.
ptRPN10-11K	pGEMT-RPN10-11K/ *pRPN10-RPN10-11K*	ptRPN10, Sm/Sc	The Sm/Sc-fragment for the *RPN10* coding region from ptRPN10 was replaced by the *RPN10-11K* coding region from pGEMT-RPN10-11K.
ptRPN10-11R	pGEMT-RPN10-11R/ *pRPN10-RPN10-11R*	ptRPN10, Sm/Sc	The Sm/Sc-fragment for the *RPN10* coding region from ptRPN10 was replaced by the *RPN10-11R* coding region from pGEMT-RPN10-11R.
ptRPN10-11-pt12RR	pGEMT-RPN10-11-12RR/ *pRPN10-RPN10-11-12RR*	ptRPN10, Sm/Sc	The Sm/Sc-fragment for the *RPN10* coding region from ptRPN10 was replaced by the *RPN10-11-12RR* coding region from pGEMT-RPN10-11-12RR.
ptRPN10-10-14A5	pGEMT-RPN10-11-14A5/ *pRPN10-RPN10-10-14A5*	ptRPN10, Sm/Sc	The Sm/Sc-fragment for the *RPN10* coding region from ptRPN10 was replaced by the *RPN10-10-14A5* coding region from pGEMT-N10-11-14A5.
Intermediate *RPN13* constructs for Agrobacterium-mediated transformation			
pKS-pRPN13	Col-0 genomic DNA/*RPN13* promoter (*pRPN13*)	pBluescript-KS+, S/Sm	pRPN13-SAL: GGA**gTCgac**TTCGACAGCGACATCGAAGCAATCAG<br>pRPN13-SMA: CTC**accCggG**TGAACACTATTCAGTAAACAGAGAAATCAG<br>The amplified 1.9-kb *RPN13* promoter (including the leader exon, at least 77-bp, and the 278-bp leader intron) was restricted and cloned into pBluescript-KS+.
pKS-RPN13	pET28a-RPN13/The coding region for *RPN13* (*RPN13*)	pBluescript-KS+, Sm/Sc	AtRPN13-SMA: tc**cccggg**tggtcagtcccttATGAGTTCAAGCGAAGCGTTTCCC<br>AtRPN13-SAC: atg**gagctc**TTAACTCTCATCCATCGCATCCCTTGATTG<br>The coding region for *RPN13* was amplified from pET28a-RPN13[c] and cloned into pBluescript-KS+.
p1239-RPN13-HA3	pET28a-RPN13/*RPN13-HA3*	p1239, Sm/Nc	AtRPN13-SMA: tc**cccggg**tggtcagtcccttATGAGTTCAAGCGAAGCGTTTCCC<br>AtRPN13-NCO: AAG**ccatgg**TCTCATCCATCGCATCCCTTGATTG<br>The coding region for *RPN13* was amplified from pET28a-RPN13[c] and cloned into p1239[d] to create a coding region for HA3-tagged RPN13 (*RPN13-HA3*).
pKS-RPN13-HA3	p1239-RPN13-HA3/*RPN13-HA3*	pBluescript-KS+, Sm/Sc	AtRPN13-SMA: tc**cccggg**tggtcagtcccttATGAGTTCAAGCGAAGCGTTTCCC<br>p1239-SAC: gta**gagctc**ttaGTGGACGCCTCTAGAGGAACGTAG<br>The coding region for *RPN13-HA3* was amplified from p1239-RPN13-HA3 and cloned into pBluescript-KS+.
pKS-RPN13-R67	pET28a-RPN13-R67/*RPN13-R67*	pBluescript-KS+, Sm/Sc	AtRPN13-SMA: tc**cccggg**tggtcagtcccttATGAGTTCAAGCGAAGCGTTTCCC<br>AtRPN13-SAC: atg**gagctc**TTAACTCTCATCCATCGCATCCCTTGATTG<br>The coding region for *RPN13-R67* from pET28a-RPN13-R67[c] was amplified and cloned into pBluescript-KS+.
pKS-RPN13-Q70	pET28a-RPN13-Q70/ *RPN13-Q70*	pBluescript-KS+, Sm/Sc	AtRPN13-SMA: tc**cccggg**tggtcagtcccttATGAGTTCAAGCGAAGCGTTTCCC<br>AtRPN13-SAC: atg**gagctc**TTAACTCTCATCCATCGCATCCCTTGATTG<br>The coding region for *RPN13-Q70* from pET28a-RPN13-Q70[c] was amplified and cloned into pBluescript-KS+.
pKS-RPN13-A1A2	AD-RPN13-A1A2/ *RPN13-A1A2*	pBluescript-KS+, Sm/Sc	AtRPN13-SMA: tc**cccggg**tggtcagtcccttATGAGTTCAAGCGAAGCGTTTCCC<br>AtRPN13-SAC: atg**gagctc**TTAACTCTCATCCATCGCATCCCTTGATTG<br>The coding region for *RPN13-A1A2* from AD-RPN13-A1A2 was amplified and cloned into pBluescript-KS+.
ptN10p-RPN13-HA3	pKS-RPN13-HA3/ *RPN13-HA3*	ptRPN10, Sm/Sc	The coding region for *RPN10* in ptRPN10 was replaced by the coding region for *RPN13-HA3* from pKS-RPN13-HA3.
ptN13p-RPN13-HA3	ptN10p-RPN13-HA3/*pRPN13-RPN13-HA3*	ptN10p-RPN13-HA3, S/Sm	The S/Sm-fragment for the *RPN10* promoter in ptN10p-RPN13-HA was replaced by the 1.9-kb *RPN13* promoter from pKS-pRPN13.
Final *RPN13* constructs for Agrobacterium-mediated transformation			
ptRPN13	pKS-RPN13/*pRPN13-RPN13*	ptN13p-AtRPN13-HA3, Sm/Sc	The Sm/Sc-fragment for the *RPN13-HA3* coding region from ptN13p-AtRPN13-HA was replaced by the *RPN13* coding region from pKS-RPN13.
ptRPN13-R67	pKS-RPN13-R67/ *RPN13-R67*	ptN13p-AtRPN13-HA3, Sm/Sc	The Sm/Sc-fragment for the *RPN13-HA3* coding region from ptN13p-AtRPN13-HA was replaced by the *RPN13-R67* coding region from pKS-RPN13-R67.
ptRPN13-Q70	pKS-RPN13-Q70/ *RPN13-Q70*	ptN13p-AtRPN13-HA3, Sm/Sc	The Sm/Sc-fragment for the *RPN13-HA3* coding region from ptN13p-AtRPN13-HA was replaced by the *RPN13-Q70* coding region from pKS-RPN13-Q70.
ptRPN13-A1A2	pKS-RPN13-A1A2/ *RPN13-A1A2*	ptN13p-AtRPN13-HA3, Sm/Sc	The Sm/Sc-fragment for the *RPN13-HA3* coding region from ptN13p-AtRPN13-HA was replaced by the *RPN13-A1A2* coding region from pKS-RPN13-A1A2.

Intermediate and final bait constructs for GST pull-down experiments			
T-AtRPN2a-11	cDNA library CD4-16/ *RPN2a* cDNA	pGEMT	R-long: GGAAACAGCTATGACCATGATTACGCCAAG<br>AtRPN2A-11: CTCTCTGCCTTTTGTCTTTCAACAGAAACC (45-bp down-stream of stop codon)<br>The cDNA covering full-length coding region for *RPN2a* was amplified and cloned directly into pGEMT.
GST-RPN2a	T-AtRPN2a-11/*GST-RPN2a*	pET42a, Sc/N	AtRPN2a-SAC: TCT**gagctc**ATGGCGACACCCATGGTTAGT<br>AtRPN2a-NOT: ATG**gcggccgc**TCATGAAGCGTATTCAAAGGCTTG<br>The coding region for RPN2a was amplified, restricted, and cloned into pET42a.
pSK:AtRPN2b	cDNA library CD4-16/ *RPN2b* cDNA (6-codons short at 3' end)	pBluescript-SK+, Rv	AtRPN2bNde: GCC**catATG**GTTAGTTCCGCCGGTGGGTTACTG<br>AtRPN2bSal: AGC**GTcgaC**AAAGGCCTGCGGAGGTTGTGGCTCGTC (located 6-bp upstream of stop codon)<br>The coding region (6-codon truncated at 3' end) for *RPN2b* was amplified, restricted, and directly cloned into pBluescript-SK+.
GST-RPN2b	pSK:AtRPN2b/ *GST-RPN2b*	pET42a, Sc/X	AtRPN2b-Sac: cc**gaGctc**ATGGCCGCCGCCATGGTTAGTTCC<br>AtRPN2b-Xho-ex: gg**ctcgAg**TCATGGAGAAGCGTATTCAAAGGCCTGCGGAGGTTGTGGCTC<br>The full-length coding region for *RPN2b* was amplified, restricted, and cloned into pET42a; the sequence for the last six C-terminal codons including the stop codon for *RPN2b* (grey-shaded) was added on the 3' primer AtRPN2b-XHOex to restore the full-length coding sequence for *RPN2b*.
GST-RPN13	First strand cDNAs/*GST-RPN13*	pET42a, B/X	RPN13-Bam: GTG**Ggatcc**ATGAGTTCAAGCGAAGCGTTTCCCGTG<br>RPN13-Xho: GAA**ctcGAg**AAGATTAACTCTCATCCATCGCATCCCTTG<br>The coding region for Arabidopsis *RPN13* was amplified, restricted and cloned into pET42a.
GST-RPN13-A47	GST-RPN13/*GST-RPN13-A47*	GST-RPN13	RPN13-A47-top: CTAGAGGTGACGAGGGA**gcG**ATTCATTTCCAGTGGCTTG<br>RPN13-A47-bot: CAAGCCACTGGAAATGAAT**cgC**TCCCTCGTCACCTCTAG<br>The codon for L47 of *RPN13* was site-directed mutagenized.
GST-RPN13-R67	GST-RPN13/*GST-RPN13-R67*	GST-RPN13	RPN13-R67-top: GAAGATGATCAAATTGTT**cgC**CCAGATGAAGCTCTATTTG<br>RPN13-R67-bot: CAAATAGAGCTTCATCTGG**Gcg**AACAATTTGATCATCTTC<br>The codon for F67 of *RPN13* was site-directed mutagenized.
GST-RPN13-Q70	GST-RPN13/ GST-*RPN13-Q70*	GST-RPN13	RPN13-Q70-top: GATCAAATTGTTTTCCCAGAT**cAA**GCTCTATTTGAAAAGG<br>RPN13-Q70-bot: CCTTTTCAAATAGAGC**TTg**ATCTGGGAAAACAATTTGATC<br>The codon for E70 of *RPN13* was site-directed mutagenized.
GST-RPN13-R88	GST-RPN13/ GST-*RPN13-R88*	GST-RPN13	RPN13-R88-top: GACAGGGTGTATATTCTGAAG**cgC**AACAGTGATGACCGCAAG<br>RPN13-R88-bot: CTTGCGGTCATCACTGTT**Gcg**CTTCAGAATATACACCCTGTC<br>The codon for R88 of *RPN13* was site-directed mutagenized.
GST-RPN13-A1A2	AD-RPN13-A1A2/GST-*RPN13-A1A2*	pET42a, B/X	RPN13-Bam: GTG**Ggatcc**ATGAGTTCAAGCGAAGCGTTTCCCGTG<br>RPN13-Xho: GAA**ctcGAg**AAGATTAACTCTCATCCATCGCATCCCTTG<br>The coding region for *RPN13-A1A2* from AD-RPN13-A1A2 was amplified, restricted, and cloned into pET42a.
GST-UCH1	First strand cDNAs/*GST-UCH1*	pET42a, R/S	AtUCH1-RI: CGT**gaAttC**ATGTCTTGGCTTCCTGTAGAATCTG<br>AtUCH1-Sal: AAA**GtcGAc**TTAGGGATGGTGCTTAGCAATGAGAG<br>The coding region for *UCH1* was amplified, restricted, and cloned into pET42a.
GST-UCH2	First strand cDNAs/*GST-UCH2*	pET42a, S/X	AtUCH2-Sal: AGTA**GTcgac**TCATGTCTTGGTGCACGATTGAGTC<br>AtUCH2-Xho: CAT**ctcGAg**GTGTCTACATTCTCAAGTGGAAC<br>The coding region for *UCH2* was amplified, restricted, and cloned into pET42a.
Final prey constructs for GST pull-down experiments			
RPN2a	GST-RPN2a/ *RPN2a*	pET28a, Sc/N	The coding region for *RPN2a* was mobilized from GST-RPN2a to *SacI/NotI* sites of pET28a.
RPN2b	GST-RPN2b/ *RPN2b*	pET28a, Sc/X	The coding region for *RPN2b* was mobilized from GST-RPN2b to *SacI/XhoI* sites of pET28a.
RPN13	First strand cDNAs/*RPN13*	pET28a, B/X	RPN13-Bam: GTG**Ggatcc**ATGAGTTCAAGCGAAGCGTTTCCCGTG<br>RPN13-Xho: GAA**ctcGAg**AAGATTAACTCTCATCCATCGCATCCCTTG<br>The coding region for Arabidopsis *RPN13* was amplified, restricted and cloned into pET28a.
RPN13-A47	GST-RPN13-A47/*RPN13-A47*	pET28a, B/X	The coding region for *RPN13-A47* was mobilized from GST-RPN13-A47 to pET28a.
RPN13-R67	GST-RPN13-R67/*RPN13-R67*	pET28a, B/X	The coding region for *RPN13-R67* was mobilized from GST-RPN13-R67 to pET28a.
RPN13-Q70	GST-RPN13-Q70/*RPN13-Q70*	pET28a, B/X	The coding region for *RPN13-Q70* was mobilized from GST-RPN13-Q70 to pET28a.
RPN13-R88	GST-RPN13-R88/*RPN13-R88*	pET28a, B/X	The coding region for *RPN13-R88* was mobilized from GST-RPN13-R88 to pET28a.
UCH1	GST-UCH1/ *UCH1*	pET28a, R/S	The coding region for *UCH1* from GST-UCH1 was subcloned into pET28a.
UCH2	GST-UCH2/ *UCH2*	pET28a, S/X	The coding region for *UCH2* from GST-UCH2 was subcloned into pET28a.
BD-fusion constructs for yeast-two-hybrid analyses			
BD-RPN2a	T-AtRPN2a-11/ *BD-RPN2a*	pBD-GAL4 Cam, Sm/S	BD-AtRPN2a-SRF1: TGT**gCcCgggC**AATGGCGACACCCATGGTTAGTTC<br>BD-AtRPN2a-SAL: GAA**gTcgAc**TCATGAAGCGTATTCAAAGGCTTG<br>The coding region for *RPN2a* was amplified, restricted and cloned into pBD-GAL4 Cam.
BD-RPN2b	pSK:AtRPN2b/ *BD-RPN2b*	pBD-GAL4 Cam, Sm/S	BD-AtRPN2b-SRF: ttc**gcccGGgc**cATGGCCGCCGCCATGGTTAGTTCC<br>B/AD-AtRPN2b-SALex: Agg**gtcgAc**TCATGGAGAAGCGTATTCAAAGGCCTGCGGAGGTTGTGGCTC<br>The full-length coding region for *RPN2b* was amplified, restricted, and cloned into pBD-GAL4 Cam; the sequence for the last six C-terminal codons including the stop codon for *RPN2b* (grey-shaded) was added on the 3' primer B/AD-AtRPN2b-SALex to restore the full-length coding sequence for *RPN2b*.
BD-RPN13	RPN13/ *BD-RPN13*	pBD-GAL4 Cam, S/P	AtRPN13SAL: GAGG**gtcgac**TTATGAGTTCAAGCGAAGCGTTTCC<br>AtRPN13PST: tcG**ctgcag**TTAACTCTCATCCATCGCATCCC<br>The coding region for *RPN13* was amplified, restricted and cloned into pBD-GAL4 Cam.
BD-UCH1	First strand cDNAs/ *BD-UCH1*	pBD-GAL4 Cam, R/S	AtUCH1-RI: CGT**gaAttC**ATGTCTTGGCTTCCTGTAGAATCTG<br>AtUCH1-Sal: AAA**GtcGAc**TTAGGGATGGTGCTTAGCAATGAGAG<br>The coding region for *UCH1* was amplified, restricted and cloned into pBD-GAL4 Cam.

BD-UCH2	First strand cDNAs/ *BD-UCH2*	pBD-GAL4 Cam, S/P	AtUCH2-Sal: AGTAGTcgacTCATGTCTTGGTGCACGATTGAGTC<br>AtUCH2-Pst: GAActgcAGAGAAGTGTCTACATTCTCAAGTGG<br>The coding region for *UCH2* was amplified, restricted and cloned into pBD-GAL4 Cam.
BD-UCH2-CΔ1	UCH2/ *BD-UCH2-CΔ1*	pBD-GAL4 Cam, S/P	AtUCH2-SAL: AGTAGTcgacTCATGTCTTGGTGCACGATTGAGTC<br>AtUCH2-CΔ1-PST: GGCCTgCagtcaCAGAGGTTTCAACTGTTTCTTCTC<br>The coding region for *UCH2-CΔ1* was amplified, restricted and cloned into pBD-GAL4 Cam.
BD-UCH2-A1	BD-UCH2/ *BD-UCH2-A1*	pBD-GAL4 Cam, S/P	AtUCH2-A1-top: GAAACAGTTGAAACCTCTGATTGcGgcGGCCgcGgcACAGAAAACAGAAAGTTCCAC<br>AtUCH2-A1-bot: GTGGAACTTTCTGTTTTCTGTgcCgcGGCCgcCgCAATCAGAGGTTTCAACTGTTTC<br>The codons in A1 region of *UCH2* were site-directed mutagenized and the coding region for *UCH2-A1* was remobilized.
BD-UCH2-A2	BD-UCH2/ *BD-UCH2-A2*	pBD-GAL4 Cam, S/P	AtUCH2-A2-top: CTGATTGAGAAGGCCAAGAAAgcGgcAgCAGcAAGTTCCACTTGAGAATGTAGAC<br>AtUCH2-A2-bot: GTCTACATTCTCAAGTGGAACTTgCTGcTgcCgcTTTCTTGGCCTTCTCAATCAG<br>The codons in A2 region of *UCH2* were site-directed mutagenized and the coding region for *UCH2-A2* was remobilized.
BD-UCH2-A3	BD-UCH2/ *BD-UCH2-A3*	pBD-GAL4 Cam, S/P	AtUCH2-A3-top: GGCCAAGAAACAGAAAACAGAAgcTgCCgCTTGAGAATGTAGACACTTCTC<br>AtUCH2-A3-bot: GAGAAGTGTCTACATTCTCAAGcGGcAgcTTCTGTTTTCTGTTTCTTGGCC<br>The codons in A3 region of *UCH2* were site-directed mutagenized and the coding region for *UCH2-A3* was remobilized.
BD-UCH2-A1-2	BD-UCH2-A1/ *BD-UCH2-A1-2*	pBD-GAL4 Cam, S/P	A1A2-top: CTGATTGcGgcGGCCgcGgcAgcGgcAgCAGcAAGTTCCACTTGAGAATGTAGAC<br>A1A2-bot: GTCTACATTCTCAAGTGGAACTTgCTGcTgcCgcTgcCgcGGCCgcCgCAATCAG<br>The codons in A2 region of *UCH2-A1* were site-directed mutagenized and the coding region for *UCH2-A1-2* was remobilized.
BD-UCH2-A2-3	BD-UCH2-A3/ *BD-UCH2-A2-3*	pBD-GAL4 Cam, S/P	A2A3-top: CTGATTGAGAAGGCCAAGAAAgcGgcAgCAGcAgcTgCCgCTTGAGAATGTAGAC<br>A2A3-bot: GTCTACATTCTCAAGcGGcAgcTgCTGcTgcCgcTTTCTTGGCCTTCTCAATCAG<br>The codons in A2 region of *UCH2-A3* were site-directed mutagenized and the coding region for *UCH2-A2-3* was remobilized.
BD-UCH2-A1/A3	BD-UCH2-A1/ *BD-UCH2-A1/A3*	pBD-GAL4 Cam, S/P	A1A3-top: GGCCgcGgcACAGAAAACAGAAgcTgCCgCTTGAGAATGTAGAC<br>A1A3-bot: GTCTACATTCTCAAGcGGcAgcTTCTGTTTTCTGTgcCgcGGCC<br>The codons in A3 region of *UCH2-A1* were site-directed mutagenized and the coding region for *UCH2-A1/A3* was remobilized.
BD-UCH2-A1-3	BD-UCH2-A1/A3/ *BD-UCH2-A1-3*	pBD-GAL4 Cam, S/P	A1-3-top: CTGATTGcGgcGGCCgcGgcAgcGgcAgCAGcAgcTgCCgCTTGAGAATGTAGAC<br>A1-3-bot: GTCTACATTCTCAAGcGGcAgcTgCTGcTgcCgcTgcCgcGGCCgcCgCAATCAG<br>The codons in A2 region of *UCH2-A1/A3* were site-directed mutagenized and the coding region for *UCH2-A1-3* was remobilized.
BD-UCH1^{2C}	UCH1/ *BD-UCH1^{2C}*	pBD-GAL4 Cam, R/S	AtUCH1-RI: CGTgaAttCATGTCTTGGCTTCCTGTAGAATCTG<br>AtUCH1-2C-SAL: TCTgtcgaCTCAAGTGGAACTTTCTGTTTTCTGTTTCTTGGCCTTCTCAATGAGAGGTTTCAGCTTCTTCTTGTCAG<br>The coding region for *UCH1^{2C}* was amplified, restricted and cloned into pBD-GAL4 Cam. The grey-shaded region was designed according to *UCH2* 3' end allowing replacement of the last five *UCH1* codons with the last 12 *UCH2* codons.
BD-UCH1^{2CA2}	BD-UCH1^{2C}/ *BD-UCH1^{2CA2}*	pBD-GAL4 Cam, S/P	AtUCH1-2CA2-top: CTCATTGAGAAGGCCAAGAAAgcGgcAgCAGcAAGTTCCACTTGAgtcgactctag<br>AtUCH1-2CA2-bot: ctagagtcgaCTCAAGTGGAACTTgCTGcTgcCgcTTTCTTGGCCTTCTCAATGAG<br>The codons on A2 region of *UCH^{2C}* attached to *UCH1* were mutagenized and the coding region for *BD-UCH1^{2CA2}* was remobilized. The grey-shaded region was designed based on the *UCH2* C-terminal region.
BD-UCH1^{2CA3}	BD-UCH1^{2C}/ *BD-UCH1^{2CA3}*	pBD-GAL4 Cam, S/P	AtUCH1-2CA3-top: GCCAAGAAACAGAAAACAGAAgcTgCCgCTTGAgtcgactctagagccctatag<br>AtUCH1-2CA3-bot: ctatagggctctagagtcgaCTCAAGcGGcAgcTTCTGTTTTCTGTTTCTTGGC<br>The codons on A3 region of *UCH^{2C}* attached to *UCH1* were mutagenized and the coding region for *BD-UCH1^{2CA3}* was remobilized. The grey-shaded region was designed based on the *UCH2* C-terminal region.
Intermediate and final AD-fusion constructs for yeast-two-hybrid analyses			
AD-RPN2a-3'	T-AtRPN2a-11/ *AD-RPN2a-3'*	pAD-GAL4-2.1, P/Bg	AD-AtRPN2a-PST-u: TGGAGAGACATCTATTCAGCTGACAC<br>AD-AtRPN2a-BGL: GAAAgAtctTCATGAAGCGTATTCAAAGGCTTG<br>A 2014-bp 3'-half coding region of *RPN2a* containing a unique *Pst*I site was amplified, restricted and cloned into pAD-GAL4-2.1.
AD-RPN2a	T-AtRPN2a-11/ *AD-RPN2a*	AD-RPN2a-3', S/P	AD-AtRPN2a-SAL: AGTGTcgaCCATCAATGGCGACACCCATGGTTAG<br>AD-AtRPN2a-PST-d: TCAGCAGGTCCGATTTGTTGTGGCTG<br>A 1089-bp 5'-half coding region of *RPN2a* overlapping with the 2014-bp 3'-half and containing the unique *Pst*I site was amplified, restricted and cloned into *AD-RPN2a-3'*.
AD-RPN2b	pSK:AtRPN2b/ *AD-RPN2b*	pAD-GAL4-2.1, X/S	AD-AtRPN2b_Xho: ccactcgagATGGCCGCCGCCATGGTTAGTTCC<br>B/AD-AtRPN2b-SALex: AgggtcgAcTCATGGAGAAGCGTATTCAAAGGCCTGCGGAGGTTGTGGCTC<br>The full-length coding region for *RPN2b* was amplified, restricted, and cloned into pAD-GAL4-2.1; the sequence for the last six C-terminal codons including the stop codon for *RPN2b* was added on the 3' primer B/AD-AtRPN2b-SALex to restore the full-length coding sequence for *RPN2b*.
AD-RPN13	BD-RPN13/ *AD-RPN13*	pAD-GAL4-2.1, S/P	The coding region for *RPN13* was mobilized from BD-RPN13 to *SalI/PstI* sites of pAD-GAL4-2.1.
AD-RPN13-22A	AD-RPN13/ *AD-RPN13-22A*	pAD-GAL4-2.1, S/P	RPN13-A22-top: GAGTTTCGTGCTGGGAAGgcGTCTTTGCAGGGAACAAG<br>RPN13-A22-bot: CTTGTTCCCTGCAAAGACgcCTTCCCAGCACGAAACTC<br>The M22 codon for RPN13 was site-directed mutagenized and the coding region for *RPN13-22A* was remobilized.
AD-RPN13-101A	AD-RPN13/ *AD-RPN13-101A*	pAD-GAL4-2.1, S/P	RPN13-A101-top: TTCTTCTGGATGCAGGcGCCAAGAGCTGAAGGTG<br>RPN13-A101-bot: CACCTTCAGCTCTTGGCgCCTGCATCCAGAAGAA<br>The E101 codon for RPN13 was site-directed mutagenized and the coding region for *RPN13-101A* was remobilized.
AD-RPN13-EL-AA	AD-RPN13/ *AD-RPN13-EL-AA*	pAD-GAL4-2.1, S/P	RPN13-ELAA-top: GGGGATATCTTGAAACCGGcGgcGATAATGCCATTGCTTGAG<br>RPN13-ELAA-bot: CTCAAGCAATGGCATTATCgcCgCCGGTTTCAAGATATCCCC<br>The EL codons (208-209) for RPN13 were site-directed mutagenized and the coding region for *RPN13-EL-AA* was remobilized.
AD-RPN13-KD-AA	AD-RPN13/ *AD-RPN13-KD-AA*	pAD-GAL4-2.1, S/P	RPN13-KDAA-top: GGTATAGACCCAAGTAAATACgcGTTCACAGTGGcCTCATTCCTTGAAGCACTTG<br>RPN13-KDAA-bot: CAAGTGCTTCAAGGAATGAGgCCACTGTGAACgcGTATTTACTTGGGTCTATACC<br>The KD codons (275/279) for RPN13 were site-directed mutagenized and the coding region for *RPN13-KD-AA* was remobilized.

AD-RPN13-ELKD-A4	AD-RPN13-EL-AA/*AD-RPN13-ELKD-A4*	pAD-GAL4-2.1, S/P	RPN13-KDAA-top: GGTATAGACCCAAGTAAATACgcGTTCACAGTGGcCTCATTCCTTGAAGCACTTG<br>RPN13-KDAA-bot: CAAGTGCTTCAAGGAATGAGgCCACTGTGAACgcGTATTTACTTGGGTCTATACC<br>The KD codons (275/279) for RPN13-EL-AA were site-directed mutagenized and the coding region for *RPN13-ELKD-A4* was remobilized.
AD-RPN13-CΔ1	AD-RPN13/*AD-RPN13-CΔ1*	pAD-GAL4-2.1, S/P	AtRPN13SAL: GAGGgtcgacTTATGAGTTCAAGCGAAGCGTTTCC<br>AtRPN13-CΔ1-PST: CATCtgcagttaTGATTGCGTTGAAACCGAGTC<br>The coding region for *RPN13-CΔ1* was amplified, restricted, and cloned into pAD-GAL4-2.1.
AD-RPN13-CΔ2	AD-RPN13/*AD-RPN13-CΔ2*	pAD-GAL4-2.1, S/P	AtRPN13SAL: GAGGgtcgacTTATGAGTTCAAGCGAAGCGTTTCC<br>AtRPN13-CΔ2-PST: TTTctgcaGtTaTATACCAAACTGAATCAAATCTATT<br>The coding region for *RPN13-CΔ2* was amplified, restricted, and cloned into pAD-GAL4-2.1.
AD-RPN13-CΔ3	AD-RPN13/*AD-RPN13-CΔ3*	pAD-GAL4-2.1, S/P	AtRPN13SAL: GAGGgtcgacTTATGAGTTCAAGCGAAGCGTTTCC<br>AtRPN13-CΔ3-PST: TTGctGcAgttaAGGGCTCTGCAACAACTCCAG<br>The coding region for *RPN13-CΔ3* was amplified, restricted, and cloned into pAD-GAL4-2.1.
AD-RPN13-CΔ4	AD-RPN13/*AD-RPN13-CΔ4*	pAD-GAL4-2.1, S/P	AtRPN13SAL: GAGGgtcgacTTATGAGTTCAAGCGAAGCGTTTCC<br>AtRPN13-CΔ4-PST: CAActGCAgTtaCAACTCCGGTTTCAAGATATC<br>The coding region for *RPN13-CΔ4* was amplified, restricted, and cloned into pAD-GAL4-2.1.
AD-RPN13-CΔ5	AD-RPN13/*AD-RPN13-CΔ5*	pAD-GAL4-2.1, S/P	AtRPN13SAL: GAGGgtcgacTTATGAGTTCAAGCGAAGCGTTTCC<br>AtRPN13-CΔ5-PST: ACCcTgcagTTaATCTCCAGCAATACCTACAGG<br>The coding region for *RPN13-CΔ5* was amplified, restricted, and cloned into pAD-GAL4-2.1.
AD-RPN13-CΔ6	AD-RPN13/*AD-RPN13-CΔ6*	pAD-GAL4-2.1, S/P	AtRPN13SAL: GAGGgtcgacTTATGAGTTCAAGCGAAGCGTTTCC<br>AtRPN13-CΔ6-PST: AGACtgcagTtaTACAACCAAGTTTCCCGCTCTAG<br>The coding region for *RPN13-CΔ6* was amplified, restricted, and cloned into pAD-GAL4-2.1.
AD-RPN13-A1	AD-RPN13/*AD-RPN13-A1*	pAD-GAL4-2.1, S/P	AtRPN13A1-top: GTTGTTGCAGAGCCCTCCTgcTgcTgcAgcAGTAGATGCATTTACCTATG<br>AtRPN13A1-bot: CATAGGTAAATGCATCTACTgcTgcAgcAgcAGGAGGGCTCTGCAACAAC<br>The codons in A1 region of *RPN13* were site-directed mutagenized and the coding region for *RPN13-A1* was remobilized.
AD-RPN13-A2	AD-RPN13/*AD-RPN13-A2*	pAD-GAL4-2.1, S/P	AtRPN13A2-top: CCTCCTTTTCGTCAACAAGcAGcTGCAggTgCCTATGTACTCCGCACAGG<br>AtRPN13A2-bot: CCTGTGCGGAGTACATAGGcAccTGCAgCTgCTTGTTGACGAAAAGGAGG<br>The codons in A2 region of *RPN13* were site-directed mutagenized and the coding region for *RPN13-A2* was remobilized; grey-shaded is a Gly codon.
AD-RPN13-A3	AD-RPN13/*AD-RPN13-A3*	pAD-GAL4-2.1, S/P	AtRPN13A3-top: CAAGTAGATGCATTTACCgcTGcAgcCgcCgCAGGACAAATAGATTTGACTC<br>AtRPN13A3-bot: GAGTCAAATCTATTTGTCCTGcGgcGgcTgCAgcGGTAAATGCATCTACTTG<br>The codons in A3 region of *RPN13* were site-directed mutagenized and the coding region for *RPN13-A3* was remobilized.
AD-RPN13-A4	AD-RPN13/*AD-RPN13-A4*	pAD-GAL4-2.1, S/P	AtRPN13A4-top: CCTATGTACTCCGCACAGGAgcAgcAGcTgcGACTCAGTTTGGTATAGACC<br>AtRPN13A4-bot: GGTCTATACCAAACTGAGTCgcAgCTgcTgcTCCTGTGCGGAGTACATAGG<br>The codons in A4 region of *RPN13* were site-directed mutagenized and the coding region for *RPN13-A4* was remobilized.
AD-RPN13-A5	AD-RPN13/*AD-RPN13-A5*	pAD-GAL4-2.1, S/P	AtRPN13A5-top: CCGCACAGGACAAATAGATTTGgCTgcGgcTGGTgcAGACCCAAGTAAATACAAGTTC<br>AtRPN13A5-bot: GAACTTGTATTTACTTGGGTCTgcACCAgcCgcAGcCAAATCTATTTGTCCTGTGCGG<br>The codons in A5 region of *RPN13* were site-directed mutagenized and the coding region for *RPN13-A5* was remobilized.
AD-RPN13-A4-5	AD-RPN13-A5/*AD-RPN13-A4-5*	pAD-GAL4-2.1, S/P	RPN13-A4-5 top: CCTATGTACTCCGCACAGGAgcAgcAGcTgcGgCTgcGgcTGGTgcAGACC<br>RPN13-A4-5 bot: GGTCTgcACCAgcCgcAGcCgcAgCTgcTgcTCCTGTGCGGAGTACATAGG<br>The codons in A4 region for RPN13-A5 were site-directed mutagenized and the coding region for *RPN13-A4-5* was remobilized.
AD-RPN13-A3-5	AD-RPN13-A4-5/*AD-RPN13-A3-5*	pAD-GAL4-2.1, S/P	RPN13-A3-5 top: CAACAAGTAGATGCATTTACCgcTGcAgcCgcCgCAGGAgcAgcAGcTgcGgCTgc<br>RPN13-A3-5 bot: gcAGcCgcAgCTgcTgcTCCTGcGgcGgcTgCAgcGGTAAATGCATCTACTTGTTG<br>The codons in A3 region for RPN13-A4-5 were site-directed mutagenized and the coding region for *RPN13-A3-5* was remobilized.
AD-RPN13-A2-5	AD-RPN13-A3-5/*AD-RPN13-A2-5*	pAD-GAL4-2.1, S/P	RPN13-A2-5 top: CCTCCTTTTCGTCAACAAGcAGcTGCAgcTgCCgcTGcAgcCgcCgCAGG<br>RPN13-A2-5 bot: CCTGcGgcGgcTgCAgcGGcAgcTGCAgCTgCTTGTTGACGAAAAGGAGG<br>The codons in A2 region for RPN13-A3-5 were site-directed mutagenized and the coding region for *RPN13-A2-5* was remobilized.
AD-RPN13-A1-2	AD-RPN13-A2/*AD-RPN13-A1-2*	pAD-GAL4-2.1, S/P	RPN13-A1-2 top: GTTGTTGCAGAGCCCTCCTgcTgcTgcAgcAGcAGcTGCAggTgCCTATG<br>RPN13-A1-2 bot: CATAGGcAccTGCAgCTgCTgcTgcAgcAgcAGGAGGGCTCTGCAACAAC<br>The codons in A1 region for RPN13-A2 were site-directed mutagenized and the coding region for *RPN13-A1-2* was remobilized; grey-shaded is a Gly codon.
AD-RPN13-A1-3	AD-RPN13-A1-2/*AD-RPN13-A1-3*	pAD-GAL4-2.1, S/P	RPN13-A1-3 top: gcAGcAGcTGCAggTgCCgcTGcAgcCgcCgCAGGACAAATAGATTTGACTC<br>RPN13-A1-3 bot: GAGTCAAATCTATTTGTCCTGcGgcGgcTgCAgcGGcAccTGCAgCTgCTgc<br>The codons in A3 region for RPN13-A1-2 were site-directed mutagenized and the coding region for *RPN13-A1-3* was remobilized; grey-shaded is a Gly codon.
AD-RPN13-A1-4	AD-RPN13-A1-3/*AD-RPN13-A1-4*	pAD-GAL4-2.1, S/P	RPN13-A1-4 top: CCgcTGcAgcCgcCgCAGGAgcAgcAGcTgcGACTCAGTTTGGTATAGACC<br>RPN13-A1-4 bot: GGTCTATACCAAACTGAGTCgcAgCTgcTgcTCCTGcGgcGgcTgCAgcGG<br>The codons in A4 region for RPN13-A1-3 were site-directed mutagenized and the coding region for *RPN13-A1-4* was remobilized.
AD-RPN13-A1-5	AD-RPN13-A1-4/*AD-RPN13-A1-5*	pAD-GAL4-2.1, S/P	RPN13-A1-5 top: CgcCgCAGGAgcAgcAGcTgcGgCTgcGgcTGGTgcAGACCCAAGTAAATACAAGTTC<br>RPN13-A1-5 bot: GAACTTGTATTTACTTGGGTCTgcACCAgcCgcAGcCgcAgCTgcTgcTCCTGcGgcG<br>The codons in A5 region for RPN13-A1-4 were site-directed mutagenized and the coding region for *RPN13-A1-5* was remobilized.
AD-UCH1	GST-UCH1	pAD-GAL4-2.1, R/S	The fragment for UCH1 coding region from GST-UCH1was subcloned into pAD-GAL4-2.1.
AD-UCH2	BD-UCH2	pAD-GAL4-2.1, S/P	The fragment for UCH2 coding region from BD-UCH2 was subcloned into pAD-GAL4-2.1.

[a]pBI121-related constructs were originally constructed for transformation. However, due to 35S promoter could not drive proper RPN10 expression, these constructs were used as intermediate source clones; ptN10p-RPN13-HA3, an RPN10 promoter (pN10)-driven construct.
[b]Restriction sites: B, *Bam*HI; Bg, *Bgl*II; N, *Not*I; Nc, *Nco*I; Nd, *Nde*I; P, *Pst*I; R, *Eco*RI; Rv, *Eco*RV; S, *Sal*I; Sc, *Sac*I; Sm, *Sma*I; X, *Xho*I.
[c]pET28a-RPN13, pET28a-RPN13-R67, and pET28a-RPN13-Q70 have been described previously [5].
[d]p1239 is a source vector for HA3-tag, which has been described previously [10].
